# Supplementary material for: NKG2A Expression among CD8 Cells Is Associated with COVID-19 Progression in Hypertensive Patients: Insights from the BRACE CORONA Randomized Trial
Source: J Clin Med. 2022 Jun 27;11(13):3713. doi: 10.3390/jcm11133713 (PMC9267446; doi:10.3390/jcm11133713)
Supplement: Supplementary file 1 [file jcm-11-03713-s001.zip › jcm-1772892-supplementary.pdf]

Journal of
Clinical Medicine

MDPI

Supplementary Materials

1. Supplementary Data

1.1. Inclusion Criteria

- Patients aged ≥ 18 years and hospitalized with confirmed diagnoses of 2019 coronavirus disease (COVID-19) who were using angiotensin receptor blockers or angiotensin-converting enzyme inhibitors.
- Ability of the patient (or legal representative) to provide informed consent in accordance with the International Council for Harmonization of Technical Requirements for Pharmaceuticals for Human Use Good Clinical Practice guidelines and local legislation and/or regulations.

1.2. Exclusion Criteria

- Hospitalization due to decompensated heart failure in the last 12 months.
- Use of more than three anti-hypertensive drugs.
- Use of sacubitril/valsartan.
- Requirement for mechanical ventilation.
- Hemodynamic instability in the 24 hours before COVID-19 diagnosis confirmation, acute renal failure, or shock.

2. Supplementary Tables and Figures

2.1. Supplementary Tables

Table S1. Disease severity according to modified WHO scores *.

Patient State	Descriptor	Score
Uninfected	No clinical or virological evidence of infection	0
Ambulatory	No limitation of activities	1
	Limitation of activities	2
Hospitalized—mild disease	Hospitalized, no oxygen therapy	3
	Oxygen by mask or nasal prongs	4
	Non-invasive ventilation or high-flow oxygen	5
Hospitalized—severe disease	Intubation and mechanical ventilation	6
	Ventilation + vasopressors, dialysis, or ECMO	7
Dead	Death	8

* Modified WHO Ordinal Scale for Clinical Improvement. ECMO, extracorporeal membrane oxygenation; WHO, World Health Organization. Modified from WHO R&D blueprint: COVID-19 Therapeutic Trial Synopsis [22].

Table S2. Antibodies used for cell staining *.

Marker	Fluorochrome	Maker	PartNum
CD3	APC	Exbio	1A-514-T100
CD4	PerCP-Cy5.5	BD	341654/341653
CD8	PE-Cy7	Exbio	T7-207-T100
CD14	APC-H7	Exbio	T4-293-T100
CD16	PacB	BioLegend	302032
CD19	PE-Cy7	Beckman Coulter	IM3628
CD38	APC-H7	Exbio	T4-366-T100
CD45	PacO	Exbio	PO-684-T100
CD56	PerCP-Cy5.5	BioLegend	318322
HLA-DR	PacB	BioLegend	307633
NKG2A	PE	Beckman Coulter	IM3291U
ZG		*BioLegend*	*423111*

* Aliquots of 1×10^6 cells.

Table S3. Degrees of lung involvement on admission.

Lung involvement*	Total	Oxygen saturation	
		>93%	≤93%
	n (%)	*n* (%)	*n* (%)
≤25%	93 (59.6)	82 (52.6)	**11 (7.1)**
26 to 50%	56 (35.9)	42 (26.9)	**14 (9.0)**
**>50%**	**7 (4.5)**	**2 (1.3)**	**5 (3.2)**
Total	156 (100.0)	126 (80.8)	30 (19.2)

* On computed tomography (CT) examination. Bold values indicate significant lung involvement (oxygen saturation ≤ 93% or >50% on CT).

Table S4. Progression to severe disease according to WHO scores.

WHO Score*		Frequency	
		Original Scale	Grouped
	n (%)	*n* (%)	95% CI
3 = Hospitalized with no oxygen	77 (49.4)	77 (49.4)	41.6–57.2%
4 = Hospitalized + oxygen, no ventilatory support	56 (35.9)	68 (43.6)	36.0–51.4%
5 = Hospitalized + non-invasive ventilatory support	12 (7.7)		
6 = Hospitalized + mechanical ventilation	0 (0.0)	11 (7.1)	3.8–11.9%
7 = Hospitalized + mechanical ventilation + inotropic support	8 (5.1)		
8 = Death	3 (1.9)		
Total	156 (100.0)	156 (100.0)	

* Modified WHO Ordinal Scale for Clinical Improvement.

Table S5. LOSs in the hospital and ICU.

WHO Score *		Descriptive Statistics			
		Mean (SD)	Minimum	Median	Maximum
Total (*n* = 156)	Total (*n* = 156)	5.6 (3.1)	0.0	5.0	17.0
Days of symptoms †	scores 3 to 5 (*n* = 145)	5.7 (3.1)	0.0	5.0	17.0
	scores 6 to 8 (*n* = 11)	4.4 (2.4)	0.0	4.0	9.0
LOS in hospital	Total (*n* = 156)	9.1 (6.8)	2.0	7.0	33.0
	score 3 to 5 (*n* = 145)	7.7 (4.3)	2.0	6.0	31.0
	scores 6 to 8 (*n* = 11)	27.6 (7.0)	12.0	31.0	33.0
LOS in ICU ‡	Total (*n* = 111)	7.6 (6.8)	2.0	5.0	34.0
	score 3 to 5 (*n* = 100)	5.9 (4.1)	2.0	5.0	28.0
	scores 6 to 8 (*n* = 11)	23.1 (7.3)	11.0	25.0	34.0

ICU, intensive care unit; LOS, length of stay; SD, standard deviation. * Modified WHO Ordinal Scale for Clinical Improvement; † Pre-hospitalization; ‡ 45 (28.8%) patients did not stay in the ICU.

2.2. Supplementary Figures

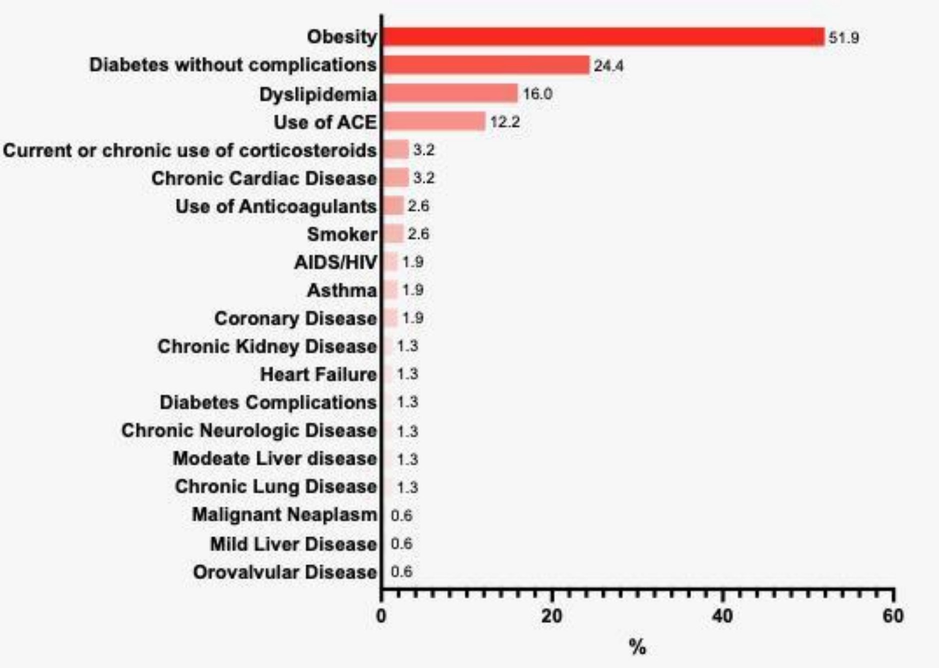

Figure S1. Comorbidities at admission (%).

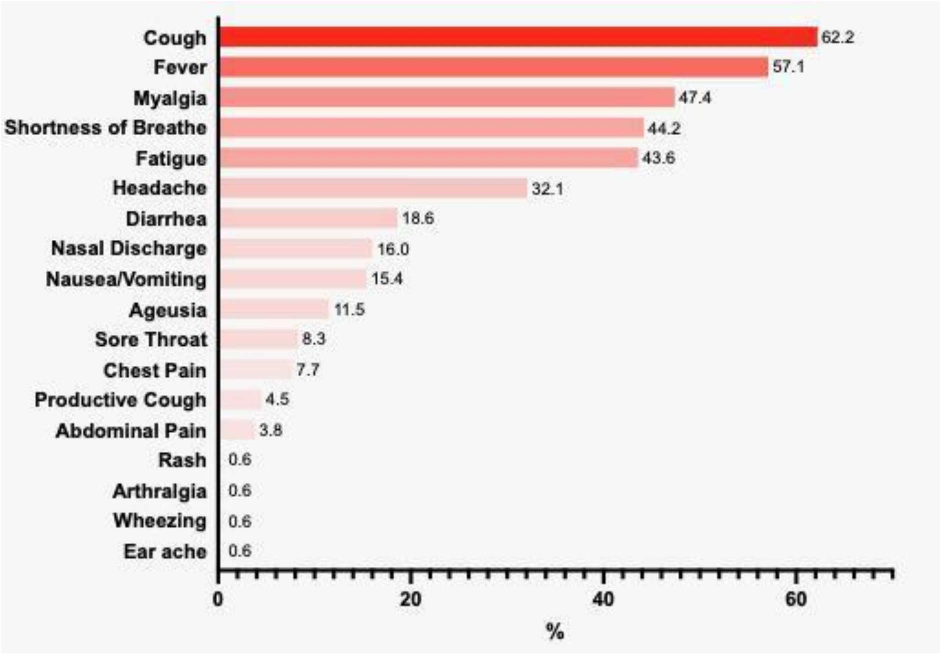

Figure S2. Symptoms at presentation (%).
